# Sub-Atomic Resolution Crystal Structures Reveal Conserved Geometric Outliers at Functional Sites

**DOI:** 10.3390/molecules24173044

**Published:** 2019-08-22

**Authors:** Saara Laulumaa, Petri Kursula

**Affiliations:** 1 Faculty of Biochemistry and Molecular Medicine & Biocenter Oulu, University of Oulu, 90014 Oulu, Finland; 2 European Spallation Source, 22100 Lund, Sweden; 3 Department of Biomedicine, University of Bergen, 5020 Bergen, Norway

**Keywords:** ultrahigh resolution, protein structure, myelin protein, fatty acid-binding protein, geometry, deuteration

## Abstract

Myelin protein 2 (P2) is a peripheral membrane protein of the vertebrate nervous system myelin sheath, having possible roles in both lipid transport and 3D molecular organization of the multilayered myelin membrane. We extended our earlier crystallographic studies on human P2 and refined its crystal structure at an ultrahigh resolution of 0.72 Å in perdeuterated form and 0.86 Å in hydrogenated form. Characteristic differences in C–H…O hydrogen bond patterns were observed between extended β strands, kinked or ending strands, and helices. Often, side-chain C–H groups engage in hydrogen bonding with backbone carbonyl moieties. The data highlight several amino acid residues with unconventional conformations, including both bent aromatic rings and twisted guanidinium groups on arginine side chains, as well as non-planar peptide bonds. In two locations, such non-ideal conformations cluster, providing proof of local functional strain. Other ultrahigh-resolution protein structures similarly contain chemical groups, which break planarity rules. For example, in Src homology 3 (SH3) domains, a conserved bent aromatic residue is observed near the ligand binding site. Fatty acid binding protein (FABP) 3, belonging to the same family as P2, has several side chains and peptide bonds bent exactly as those in P2. We provide a high-resolution snapshot on non-ideal conformations of amino acid residues under local strain, possibly relevant to biological function. Geometric outliers observed in ultrahigh-resolution protein structures are real and likely relevant for ligand binding and conformational changes. Furthermore, the deuteration of protein and/or solvent are promising variables in protein crystal optimization.

## 1. Introduction

As biological molecules work in mainly aqueous environments, details of protonation and hydrogen bonding are crucial to understanding protein function at the atomic level. Perdeuterated proteins can be used in neutron crystallography for the accurate determination of hydrogen positions and protonation states. On the other hand, it is possible that perdeuteration affects either the structure or the ligand-binding properties of the protein—or both [1]. Using ultrahigh-resolution X-ray crystallography, many hydrogen atom positions can also be determined, but relatively low levels of local disorder will already make H atoms invisible in electron density. Ultrahigh resolution also allows for the detection of structural anomalies, both in the peptide backbone and in the side chains, due to the possibility of X-ray data outweighing geometrical restraints during refinement. 

The crystal structures of only 10 different proteins have been refined at a resolution of at least 0.75 Å, and eight of these proteins are larger than 100 amino acid residues. Three human proteins have been refined at 0.75 Å or higher resolution: Cyclophilin G [2], aldose reductase [3], and the PDZ domain of syntenin [4]. Highest resolution for any protein structure has been achieved for crambin and a high-potential iron-sulphur protein, both at 0.48 Å [5,6], and the perdeuterated form of *Pyrococcus furiosus* rubredoxin has been refined at 0.59-Å resolution [7]. 

Myelin protein 2 (P2) is a myelin-specific protein of the nervous system, which acts as a peripheral membrane protein within the myelin sheath. P2 belongs to the fatty acid binding protein (FABP) family, and it is likely to function in lipid transport during myelination. P2 has a role in maintaining lipid homeostasis in the developing nervous system [8]. Mutations in the gene encoding P2 are linked to Charcot–Marie–Tooth disease [9,10,11,12,13]. We have previously determined the atomic-resolution 0.93-Å structure of human P2 (Protein Data Bank (PDB) entry 4bvm) [14], providing accurate insights into its ligand-binding determinants. The positions of many hydrogen atoms were also defined within the internal hydrogen-bonding network, and one of the fatty acid-coordinating arginine residues was shown to be in the neutral deprotonated form. 

We set out to take advantage of modern synchrotron radiation beamlines and the exceptional diffraction properties of human myelin P2 protein crystals to get an even deeper insight into the structural details of this peripheral membrane protein. The human P2 structure was refined at 0.72-Å resolution, using data collected from perdeuterated P2 (d-P2), and a number of intriguing details concerning protein structure were revealed. In addition, the structure of hydrogenated P2 (h-P2) was refined to 0.86-Å resolution using new data. The complementary use of these data gave directions for analyzing conformational abnormalities in other proteins and protein families, highlighting the conservation of conformational strain in locations possibly important for ligand binding, catalysis, and/or conformational changes. 

## 2. Results and Discussion

### 2.1. Overall Structure and Quality

**Figure 1:**
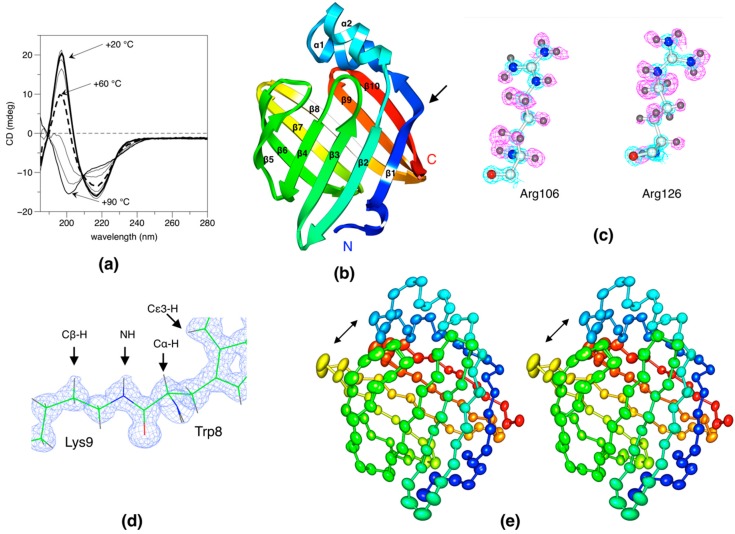
Structural properties of deuterated P2. (**a**) Circular dichroism (CD) spectra as a function of temperature from +20 to +90 °C. For clarity, only spectra at 10 °C intervals are shown. (**b**) Overall structure of human P2. The secondary structures and the termini are labelled. The arrow points at the bump in strand β1. (**c**) Quality of electron density for the two Arg residues, which coordinate the bound fatty acid. The 2F_o_–F_c_ map (blue) is contoured at 4.0 σ and the F_o_–F_c_ map (magenta) at 2.0 σ. Note how Arg106 is deprotonated, i.e., in a neutral form, as we also reported previously [14]. The maps are for hydrogenated P2 (h-P2) at 0.86 Å resolution, calculated without hydrogen atoms. (**d**) The 2F_o_–F_c_ map for perdeuterated P2 (d-P2) shows clear bumps at the positions of well-defined deuterium atoms, indicating a very high quality of the diffraction data. (**e**) Analysis of the anisotropic displacement parameters suggests open–close motions (arrows) at the portal region even in the crystal state (stereo view).

Based on CD spectra, the folding of the perdeuterated human P2 was practically identical to the hydrogenated protein (Figure 1a, Supplementary Figure S1). Thermal unfolding assays indicated d-P2 to be stable in an aqueous solution, with a melting temperature of +60 °C, which is similar to that earlier observed for h-P2 [14,15]. Upon crystallization experiments for neutron diffraction [16], large d-P2 crystals were also tested on synchrotron X-ray beamlines and observed to give sub-atomic resolution diffraction patterns [17,18].

The structure of perdeuterated human P2 was refined at 0.72-Å resolution with good statistics (Table 1). The overall structure was very similar to our earlier structure of the hydrogenated protein (Figure 1b), but the space group was different. The packing in the new space group has, in fact, caused some disorder despite the high resolution of X-ray diffraction (Supplementary Figure S2). This disorder is found inside the β barrel, around the bound fatty acid. 

In the ligand-binding cavity, the new crystal form had more disorder than observed in the previous 0.93 Å structure, and hence, less hydrogen atoms were observable, e.g., within the water-mediated hydrogen-bonding network. It is possible that deuteration weakens the interactions between the protein and the ligand/water molecules in the cavity, such that they become more dynamic. On the other hand, the protein structure was of very high quality, and fascinating details became visible at this sub-atomic resolution. 

In addition to d-P2, an ultrahigh-resolution structure of h-P2 was also refined at 0.86-Å resolution. In this case, the space group was the same as observed before [14], and despite the lower resolution, some parts of the structure—most notably the ligand and its environment (Supplementary Figure S2)—were indeed better resolved than in the d-P2 structure. The deprotonation of the fatty acid-coordinating Arg side chain is clearly visible in the electron density maps (Figure 1c). Figure 1d gives an example of the quality of electron density in d-P2, and the deuterium atoms can be seen even in the 2F_o_–F_c_ maps. When discussing details of hydrogen bonding and side chain conformation below, all the features were visible in the ultrahigh-resolution structures of both d-P2 and h-P2. 

An anisotropy analysis of the structure (Figure 1e) showed an overall directionality of atomistic anisotropy, which could be related to functional open–close movements in the protein. The opening of the β barrel is thought to be central to ligand entry and egress, and it could play a role in lipid membrane binding [18]. 

### 2.2. Side Chain-Backbone Interactions

**Figure 2:**
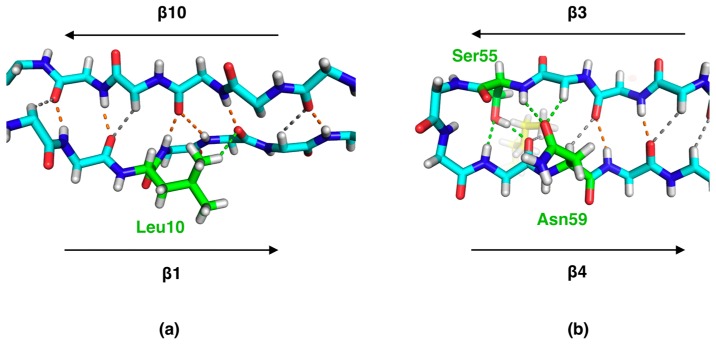
Examples of side chain involvement in β sheet hydrogen bonding. (**a**) A bump in strand β1 is observed around residue 11. A closer inspection reveals a breakdown of β sheet structure, with two NH groups H-bonding to a single carbonyl moiety and the side chain of Leu10 providing a C-H...O bond to the carbonyl group of residue 12. (**b**) Near the β3-β4 loop, which is part of the portal region and predicted to open upon ligand exchange/membrane binding, the interactions near the loop involve side chains and the fatty acid ligand (yellow), although the backbone remains in an extended β conformation. Regular hydrogen bonds are shown in orange, main-chain C-H…O bonds in gray, and bonds involving side chains in green. Only side chains participating in main-chain hydrogen bonding are visible (green).

C–H...O hydrogen bonds are generally recognized to be an integral part of protein structure, especially in β sheets. Main-chain C–H...O interactions are common features of the β sheets of P2. In addition to main-chain C–H...O bonds, a number of C–H...O hydrogen bonds between side chains and the main chain were detectable. Two examples of this in P2 are provided in Figure 2. 

**Figure 3:**
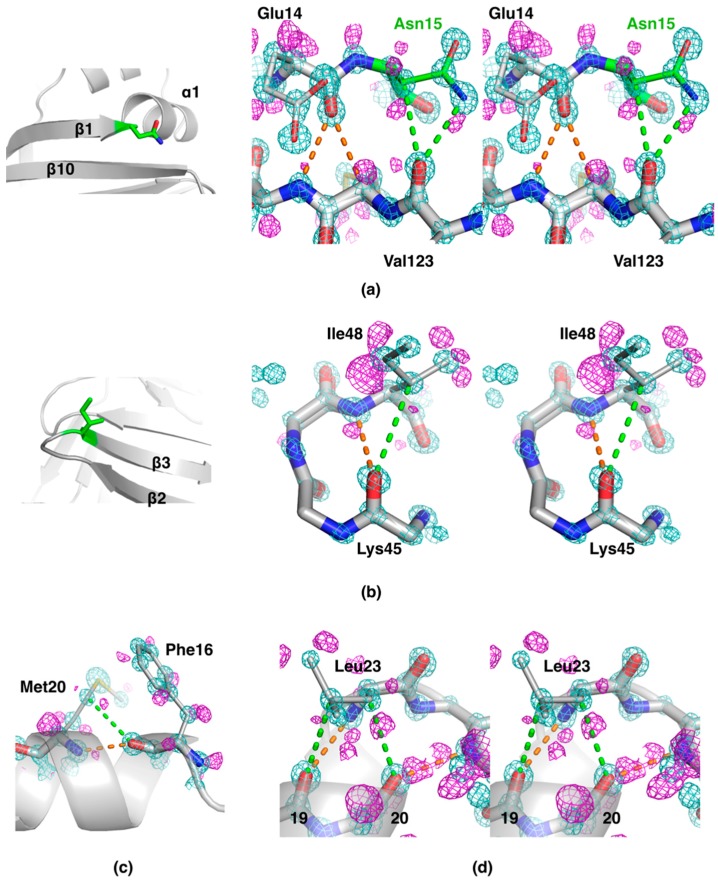
Details of protein backbone interactions from sub-atomic resolution electron density maps. Carbonyl groups in d-P2 secondary structures regularly interact with side-chain C–H groups through C–H...O hydrogen bonds. The 2F_o_–F_c_ map (cyan) in each panel is contoured at 4.0 σ, and the F_o_–F_c_ map (magenta) at 2.7, 2.5, 2.5, and 2.2 σ for panels (**a**)–(**d**), respectively. The maps were calculated after refining the structure without hydrogen atoms. Main-chain hydrogen bonds are shown in orange, and hydrogen bonds involving side chains are shown in green. (**a**) Left: Asn15 (green) is located at the C-terminal end of strand β1, just before helix α1 starts. Right: Stereo view of the interactions between the Asn15 side chain and strand β10. (**b**) The β2–β3 turn presents Cβ–H…O hydrogen bonding between Ile48 (green in the left panel) and the main-chain carbonyl of Lys45. (**c**) C–H…O bonding within helix α1, involving the side chain of Met20. (**d**) At the end of helix α1, Leu23 caps the helix through a double C–H…O interaction to the previous turn of the helix.

C–H...O bonds between side chains and main-chain carbonyl moieties were also frequently observed at the ends of secondary structure elements (Figure 3). These interactions can be considered elements of secondary structure capping in protein structures. 

At the C-terminal end of strand β1, between strands 2 and 10, the strand turns away, but the β-sheet-like interactions are continued further with strand 10 through an extended conformation of the Asn15 side chain (Figure 3a). The side chain makes N–H...O and C–H...O bonds with the carbonyl oxygen of Val123 on strand β10. The sequence alignment of all human FABP family members, 12 in total, highlights that Asn15 is fully conserved, apart from human FABP5, in which it is a glycine [14]. This observation suggests that a similar “extension” of strand β1 may be a common property of FABP family members. Another location of C–H...O bonds involving side chains concerns tight β turns (Figure 3b). While the backbone carbonyl group of residue *i* H-bonds to the NH group of residue *i+3*, it also makes a C–H...O bond to the side chain of the same residue (*i+3*) in approximately half of the turns. In the remaining cases, the carbonyl group has a different conformation and contacts a water molecule in addition to NH(*i+3*). 

In α helices, the carbonyl group at position (*i*) could be involved in a C–H...O bond to the Cβ or Cγ of a side chain in the next turn of the helix (residue *i+4* or *i+3*) (Figure 3c). For example, the side chain of Leu23 makes two C–H...O bonds, to the backbone carbonyls of residues 19 and 20, to cap helix α1 at its C-terminal end (Figure 3d). These interactions are similar to those reported earlier in helical structures [19].

### 2.3. Unconventional Side Chain Conformations in P2

**Figure 4:**
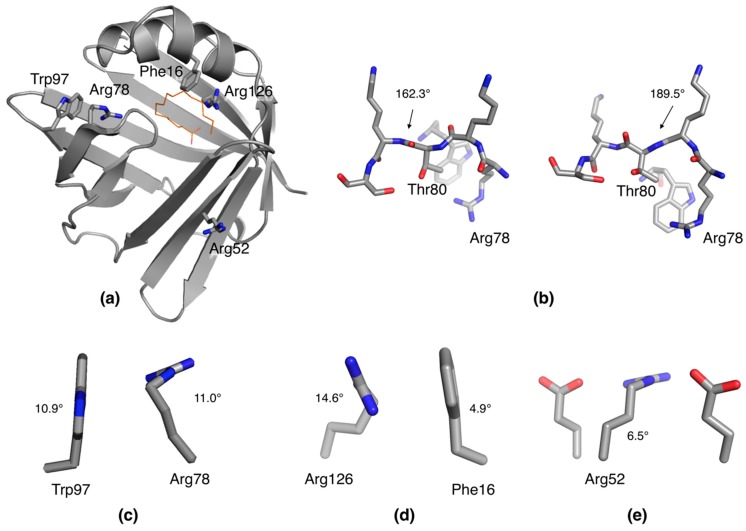
Unconventional conformations are revealed at ultrahigh resolution. (**a**) Location of the distorted side chains in the d-P2 3D structure. Note how Arg78/Trp97 and Phe16/Arg126 are in close contact. (**b**) Peptide bond distortion on both sides of Thr80 leads to its insertion deeper into the structure, interacting with Arg78 and Trp97. The ω angles for the two adjacent bent peptide bonds are shown from two slightly different views. (**c**) Packing of Trp97 against Arg78. Both side chains are strong geometric outliers due to non-planarity. (**d**) Arg126 packs against Phe16, and both residues show loss of planarity in the side chain. (**e**) Arg52 is twisted, apparently to optimize its conformation between two Glu residues.

The high resolution of the diffraction data for human P2 allows for the detection of unusual conformations in amino acid side chains. In fact, a few residues were listed as outliers by MolProbity, and a careful inspection confirmed the correctness of the model with respect to electron density. Notably, three arginine residues in P2 were non-planar in their guanidinium groups. In addition, Phe16 and Trp97 deviate significantly from planarity (Figure 4a). 

For quantifying the analysis, the following “bending angles” were measured to reflect distortion from expected planarity. For Arg residues, the torsion angle was Cδ-Nε-Cζ-Nη1 (expected 0°); for Phe, the angle was [180°-(Cβ-Cγ-Cζ)] (expected 0°); for Trp, the pseudo-torsion angle was Cβ-Cγ-Cε2-Cζ3 (expected 0°); and for Tyr, the sum of the angles [180°-(Cβ-Cγ-Cζ)] and [180°-(Cβ-Cζ-Oη)] (expected 0°). At high resolution, some peptide bonds deviate from planarity, the ω angle being different from the theoretical 180° (Figure 4b). These are the angles cited in the figures. 

Arg78 resides in the portal region, at the turn between strands β5 and β6, where it is engaged in a salt bridge with Asp76. In our previous study, Arg78 was one of the main residues interacting with the lipid membrane during coarse-grained molecular dynamics (MD) simulations [14]. In the current structure, the side chain of Arg78 is twisted such that it could make a better salt bridge; on the other hand, a non-planar aromatic residue, Trp97, is stacked edge-to-face with Arg78 (Figure 4c). Trp97 interacts with the bent guanidinium group of Arg78 with both of its rings, possibly stabilizing this unexpected conformation. This local strain may be related to conformational changes taking place when P2 binds to a membrane surface and/or the portal region opens for fatty acid release. Such opening has been observed in atomistic MD simulations and SAXS experiments [13,20]. 

Arg126 is one of the two arginine residues in P2 directly coordinating the carboxyl group of the bound fatty acid [14,15,20]. In h-P2, this residue is protonated, while Arg106 is deprotonated (Figure 1c). In the 0.72 Å d-P2 structure, the guanidinium group of Arg126 is a geometrical outlier, standing out at >8 σ deviation in the MolProbity analysis. A stacking interaction between Phe16 and Arg126 can be observed, and Phe16 is non-planar, with Cβ out of the phenyl ring plane. The guanidinium group of Arg126 is twisted so it can make face-to-face stacking with Phe16 (Figure 4d); this, on the other hand, enables it to make optimal interactions with the ligand fatty acid. Whether this strained conformation is a unique property of the liganded state cannot be answered at this time, since all crystal structures of human P2 thus far contain bound fatty acid. 

Arg52 is on the surface of P2, making salt bridges with the side chains of Glu54 and Glu61. Its guanidinium group is well-defined as non-planar. Judging from the arrangement, the twist is linked to the formation of optimal interactions to the two acidic residues, between which Arg52 is sandwiched (Figure 4e). 

Taken together, the geometric outliers observed in human P2 at high resolution can be justified based on the chemical environment. In addition, intriguing concerted bending of side chains away from planarity could be seen in cases, where the guanidium group of an Arg residue stacked against an aromatic residue. Such interactions are abundant and possibly functional in protein structures [21], and it is possible that the observations here have a more general relevance for such side chain arrangements in folded proteins. 

It should be noted that these unconventional conformations resulted in outliers in structure quality analysis, even though they were definitely based on real features of the high-quality experimental electron density map. Hence, a structure with outliers refined at ultrahigh resolution is partially beyond standard structure validation algorithms, which are based on idealized geometry libraries. Too tight validation criteria may discourage crystallographers to utilize the full information available in ultrahigh-resolution crystallographic data. 

### 2.4. Distorted Side Chain Planarity in Other High-Resolution Structures

To further assess the importance of high resolution to detect geometric outliers, selected ultrahigh-resolution protein crystal structures from the PDB were observed. In general, essentially all of such structures contain some distortions, especially the bending of aromatic side chains. For example, lysozyme is a general model protein for structural biology and biochemistry, and its 0.65 Å structure [22] reveals a number of side chains with distorted planarity. The protein with the highest-resolution structure to date, crambin, also presents a twisted guanidinium group in Arg17 [5]. In cases where ultrahigh-resolution data are available from related proteins, such features appear to be conserved within protein families, suggesting they are not merely crystallographic artifacts but true properties of the proteins, likely related to functional mechanisms due to the local strain they bring about. These aspects will be discussed below.

**Figure 5:**
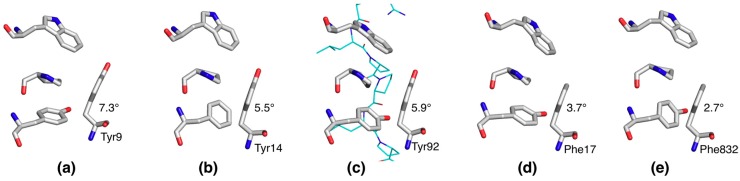
A conserved bent aromatic residue in SH3 domains. Shown above is the peptide-binding site, composed of several conserved aromatic residues, of the atomic-resolution SH3 domain structures from (**a**) yeast Bzz1, (**b**) yeast Bbc1, (**c**) chicken c-Src bound to a peptide ligand, (**d**) rat betaPIX, and (**e**) human ponsin. The bending angle of the outlier aromatic residue (bottom right) is shown for all structures.

As small proteins with a relatively large proportion of conserved aromatic residues, Src homology 3 (SH3) domains are represented in the PDB by many atomic-resolution structures. We looked at five SH3 domain structures, refined at < 1.0 Å resolution. The structures used were PDB entries 1zuu (0.97 Å), 1tg0 (0.97 Å), 4hvw (0.98 Å), 2g6f (0.92 Å), and 2o9s (0.83 Å) [23,24,25,26,27]. A conserved aromatic planarity outlier is present in all structures analyzed, close to the peptide ligand-binding site; bending was most severe for a Tyr, but also the Phe residues at this position showed the same phenomenon (Figure 5). This finding strongly suggests functional relevance for this structural anomaly in SH3 domains. 

**Figure 6:**
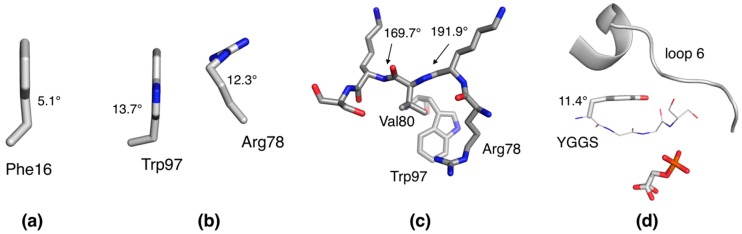
Additional geometric outliers. Compared to human P2, FABP3 shows essentially identical bending of (**a**) Phe 16, (**b**) the Trp97–Arg78 unit, and (**c**) the peptide bonds before and after residue 80. (**d**) Tyr210 is severely bent in the liganded closed structure of triosephosphate isomerase (TIM) [29].

When specifically considering the function of P2, which needs to involve opening/conformational changes in the portal region, the current data provide novel ideas for the entire FABP family. The anisotropy of P2 has clear localization and direction, and these together hint at flexibility in the portal region corresponding to eventual open/close motions (Figure 1e). Opening of the portal region is a common feature proposed for the entire FABP family, and we recently observed such conformational changes in extended MD simulations [13,20]. The concentration of bent side-chain and main-chain conformations close to each other in space, especially around Arg78, is likely to be relevant for membrane or fatty acid binding-related conformational changes in P2. Since the whole FABP family is likely to share a similar conformational change related to ligand binding, it is very important to note that essentially identical deviations from ideal geometry between P2 and FABP3 [28] can be observed in their respective ultrahigh-resolution structures (Figure 6a–c).

Finally, triosephosphate isomerase (TIM) is an enzyme, for which a huge body of research data is available, including sub-atomic resolution crystal structures in complex with active-site ligands. A conserved motif in TIM, YGGS, is relevant for conformational changes and subtrate/product binding. The Tyr residue of this motif in the 0.83 Å crystal structure of TIM from *Leishmania mexicana* [29] is severely bent, and it is likely that this strain is functionally relevant (Figure 6d). The opening of the so-called loop 6, which is in close contact with Tyr210, results in the loss of the bent conformation, which can be seen at high resolution, e.g., in the structure of TIM from *Plasmodium falciparum* [30].

### 2.5. Deuteration as a Tool for Crystallization

Proteins in biological environments are hydrogenated, apart from the rare, artificial conditions of perdeuterated protein production in cultures. Isotope effects are often observed in, e.g., enzymatic reactions, when comparing performance in normal and heavy water. Comparisons between deuterated and hydrogenated protein structures are important in assessing the reliability and relevance of neutron crystal structures to biological function, and they can be used to shed light on enzymatic catalysis. 

Together with our earlier experiments on 2′,3′-cyclic nucleotide 3′-phosphodiesterase (CNPase) [31], our study suggests that deuteration could be used as a means of crystal quality optimization, even when neutron experiments are not planned. Perdeuterated CNPase produced crystals diffracting to atomic resolution and allowed us to decipher the protonation states and orientations of water molecules in the active site; such resolutions were never reached with the same construct without deuteration [31]. Here, human P2 crystallized in deuterated form, in a D_2_O-based buffer, providing a new space group diffracting even better than the one optimized for h-P2. Several explanations can be thought of as causes of such differences; one could be shorter hydrogen bonds formed by deuterium. In addition, the recent unpublished ultrahigh-resolution structures of perdeuterated rubredoxin [7] support these findings. It might be possible to simply use D_2_O in crystallizing hydrogenated proteins when either optimizing or screening for crystallization conditions. Perhaps deuterated conditions could be used to trap enzymatic reaction intermediates in crystallo. However, deuteration can lead to changes in enzyme active-site structures per se [1]. Systematic studies on these aspects are clearly warranted. 

## 3. Materials and Methods 

### 3.1. Perdeuteration and Protein Purification

For the production of perdeuterated human P2, the facilities at the Deuteration Laboratory, ILL in Grenoble, France were used as described [16]. Hydrogenated P2 was purified using established protocols [15]. 

d-P2 was analyzed by CD spectroscopy on a Chirascan Plus instrument (Applied Photophysics, Leatherhead, UK). CD spectra were measured at 0.25 mg/mL in a 0.5 mm cuvette. Thereafter, a thermal melting experiment was also carried out using the same instrument. For comparison, the same experiments were done using h-P2, as described earlier [14]. 

### 3.2. Crystallization

Perdeuterated P2 was used for growing large crystals for neutron diffraction experiments. In the course of this procedure, a large crystal representing a new crystal form was used for X-ray data collection. This crystal was grown using hanging-drop vapour diffusion equilibrating over a mother liquour consisting of 30% PEG6000 and 0.1 M sodium citrate (pD 4.25) in 90% D_2_O. Crystal growth was carried out at +8 °C over a period of several months with intermittent feeding with fresh protein. The final size reached approximately 1.0 × 0.5 × 0.1 mm^3^. 

Hydrogenated P2 was crystallized as previously described for the 0.93-Å structure [14]. The crystal used for data collection was grown against 100 mM sodium citrate (pH 5), 24% PEG 6000.

### 3.3. Data Collection and Processing

A single large crystal of d-P2 was picked up in a loop, after a brief soak in cryoprotectant (mother liquour supplemented with 20% PEG200). The crystal was flash-cooled in liquid nitrogen. Diffraction data were collected at 100 K on the PETRAIII/DESY synchrotron radiation beamline P11 [32]. Data were processed using XDS [33]. For data collection from a h-P2 crystal with a similar procedure, beamline P13 at EMBL/DESY [34] was used. The diffraction images for both crystals can be found on the zenodo.org server [17,18]. For determining the high-resolution cutoff for both datasets, we used the criteria <I/σ(I)> ≈ 1.0 and CC_1/2_ > 30%. Recent studies have shown that in fact, diffraction data up to <I/σ(I)> ≈ 0.5 and CC_1/2_ ≈ 10% could have been included [35,36]. The Wilson and Luzzati plots for both datasets are given in Supplementary Figure S3. The Luzzati plots show that R_free_ remains under 40% in the highest-resolution shell, justifying the resolution cutoff.

### 3.4. Structure Solution and Refinement

The structure was solved by molecular replacement, using the 0.93-Å structure of P2 (PDB entry 4bvm) [14] as a search model. Refinement was carried out using phenix.refine [37], and model building was done using coot [38]. During refinement, hydrogen atoms were added (either as D or H), and all atoms except hydrogen were refined anisotropically. The structure was validated using MolProblity [39] and PARVATI [40]. Structure analyses and figure preparation were carried out using PyMOL [41], ccp4mg [42], and UCSF Chimera [43]. The coordinates and structure factors were deposited at the Protein Data Bank with entry codes 6S2M (d-P2) and 6S2S (h-P2). 

## 4. Conclusions

The refinement of the human P2 structure at 0.72-Å resolution reveals a number of features only observable at such high resolution. These include new details on main-chain interactions, side chain conformations, and different types of hydrogen bonding. However, for the accurate hydrogen/deuterium positioning of more atoms, neutron diffraction data would be required. Our earlier attempts at this produced an incomplete lower-resolution dataset [16]. On the other hand, the unexpected side chain conformations discussed above did not catch our attention during the refinement of the previous 0.93-Å structure of the hydrogenated P2 protein [14]; in retrospect, most of them can be identified in the earlier structure. Hence, for accurate descriptions of non-ideal conformations and local strain in crystal structures, it is clear that resolutions well below 1.0 Å are required. One must also have a keen eye on looking for such anomalies instead of trying to fit every residue into a geometric ideal norm. Especially at very high resolutions, intentionally loosening geometric restraints may allow automatic outlier detection algorithms to detect real outliers of side chain and main chain conformations. On the other hand, the manual inspection of key regions of interest and their fit to electron density is always worth the effort when ultrahigh-resolution data are at hand. Such data, currently only attainable through X-ray crystallography, will give novel insights into the detailed mechanisms of protein function in biological systems, even conserved across entire protein families.

## Figures and Tables

**Table 1:** Data processing and structure refinement. The values in parentheses refer to the highest-resolution shell.

Sample	d-P2	h-P2
Space group	*C*2	*P*4_1_2_1_2
Unit cell dimensions	*a* = 112.18 Å, *b* = 36.21 Å, *c* = 31.11 Å, *α* = *γ* = 90°, *β* = 97.03°	*a* = *b* = 57.93 Å, c = 101.32 Å, *α* = *β* = *γ* = 90°
Wavelength (Å)	0.7443	0.8266
Resolution range (Å)	50–0.72 (0.74–0.72)	30–0.86 (0.88–0.86)
<*I*/σ(*I*)>	15.8 (0.9)	14.7 (1.1)
*R_sym_* (%)	3.6 (121.4)	6.3 (169.9)
*R_meas_* (%)	3.9 (143.5)	6.8 (183.0)
Completeness (%)	91.0 (53.2)	94.4 (87.1)
Redundancy	4.7 (3.3)	7.2 (7.0)
CC_1/2_ (%)	99.9 (40.5)	99.8 (43.3)
Wilson *B* factor (Å^2^)	9.0	11.3
Mosaicity (°)	0.053	0.067
*R_cryst_* (%)	10.4	9.9
*R_free_* (%)	11.1	11.8
rmsd bond lengths (Å)	0.020	0.020
rmsd bond angles (°)	1.8	1.9
Average *B* factor (Å^2^); protein, ligand, solvent	10.7, 14.1, 21.4	11.0, 11.6, 21.6
Ramachandran favoured/allowed (%); Molprobity score (percentile)	100/100; 1.30 (85th)	99.2/100; 1.25 (86th)
Mean anisotropy;protein, ligand, solvent	0.41 ± 0.13, 0.41 ± 0.14, 0.40 ± 0.15	0.49 ± 0.15, 0.46 ± 0.12, 0.41 ± 0.16
PDB entry	6S2M	6S2S

## Supplementary Materials

The following are available online at https://www.mdpi.com/1420-3049/24/17/3044/s1, Figure S1: Comparison of CD spectra for d-P2 (black) and h-P2 (red)., Figure S2: Comparison of crystal packing and disorder., Figure S3: Wilson and Luzzati plots.
