# Supplementary material for: Sub-Atomic Resolution Crystal Structures Reveal Conserved Geometric Outliers at Functional Sites
Source: Molecules. 2019 Aug 22;24(17):3044. doi: 10.3390/molecules24173044 (PMC6749445; doi:10.3390/molecules24173044)
Supplement: Supplementary file 1 [file molecules-24-03044-s001.pdf]

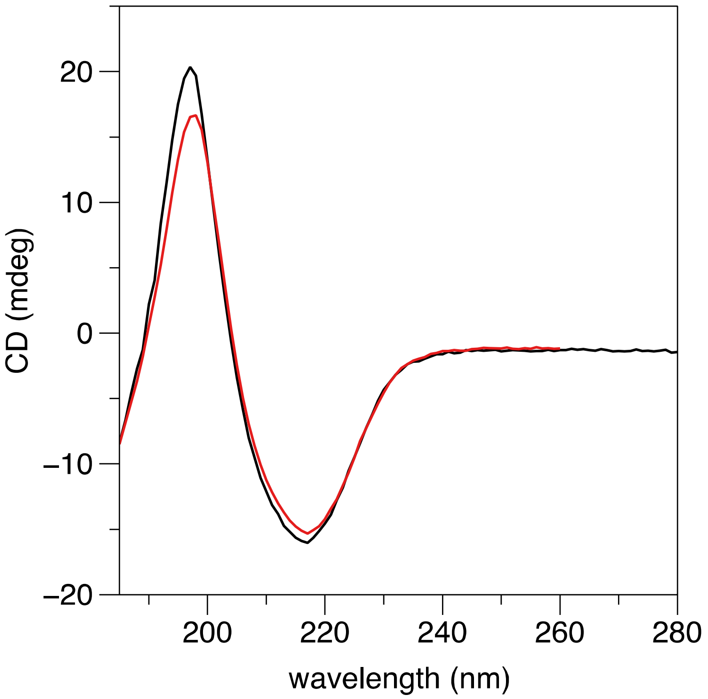

Supplementary Figure S1. Comparison of CD spectra for d-P2 (black) and h-P2 (red).

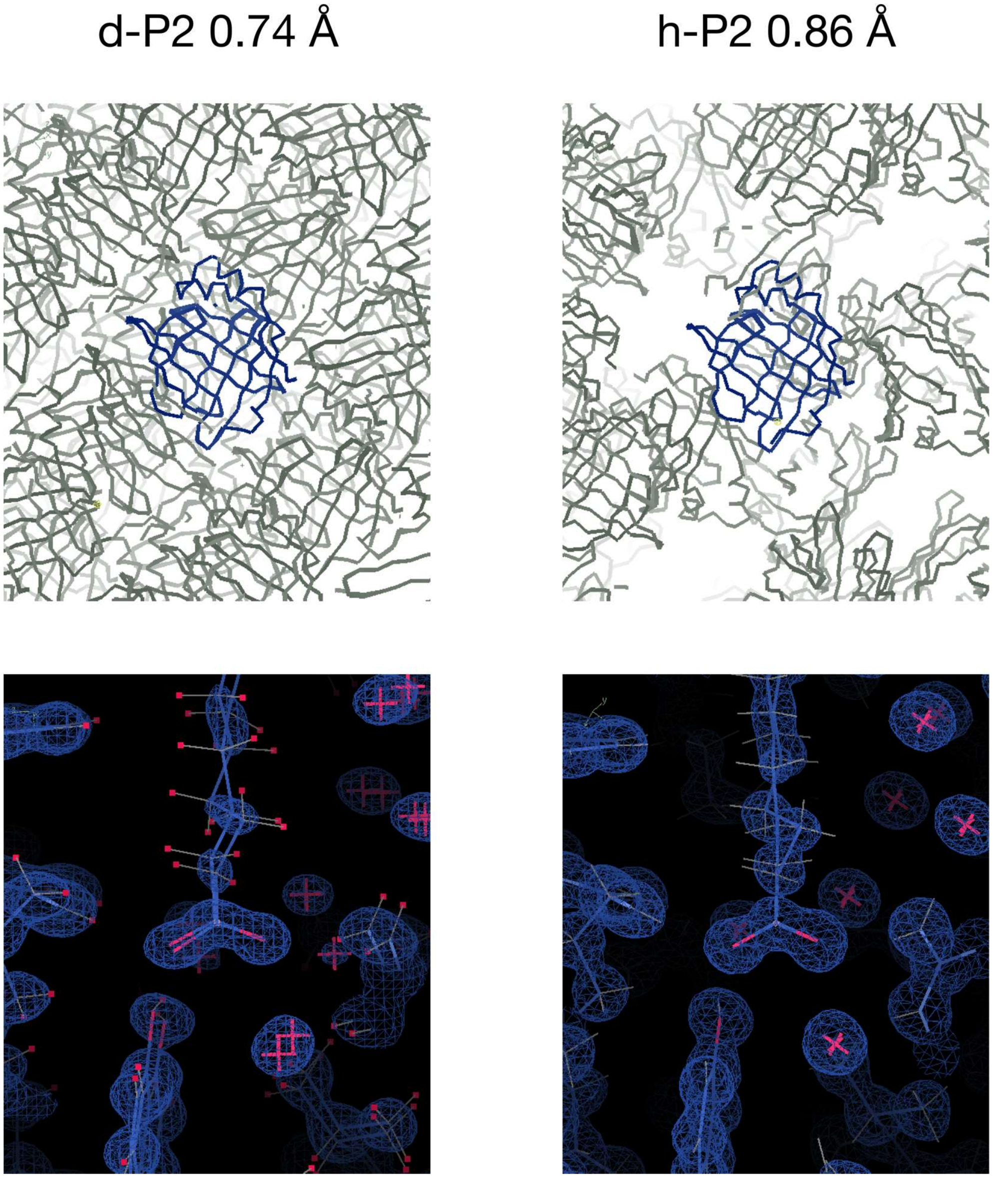

Supplementary Figure S2. Comparison of crystal packing and disorder.

Top: crystal packing visualized for the crystal forms of d-P2 and h-P2 indicate much tighter packing for d-P2. The blue molecule representing the asymmetric unit is in the same orientation in both panels.

Bottom: The binding site for the fatty acid ligand is more disordered in d-P2. The map is the final $2F_o$-F_c map at 1.5 σ level.

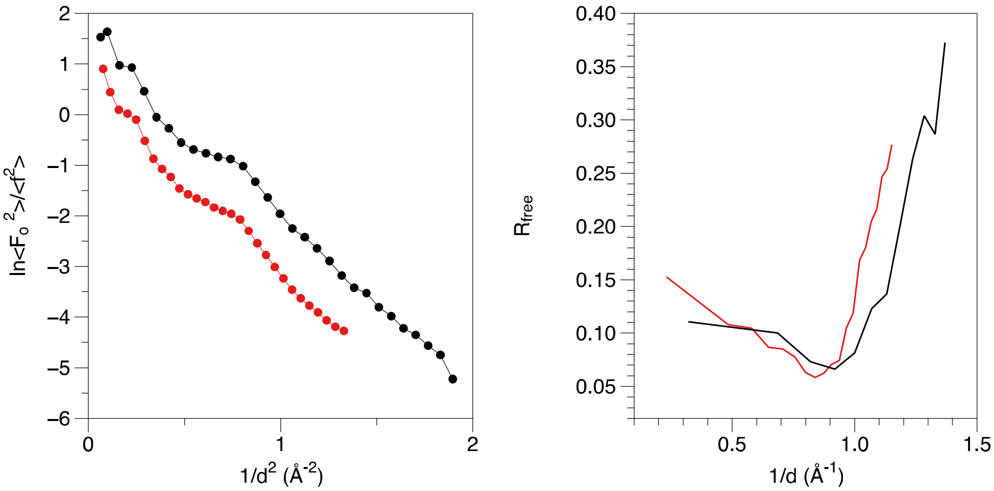

Supplementary Figure S3. Wilson (left) and Luzzati (right) plots for d-P2 (black) and h-P2 (red).
